# Chronic Pain in Inflammatory Arthritis: Mechanisms, Metrology, and Emerging Targets—A Focus on the JAK-STAT Pathway

**DOI:** 10.1155/2018/8564215

**Published:** 2018-02-07

**Authors:** Fausto Salaffi, Giovanni Giacobazzi, Marco Di Carlo

**Affiliations:** ^1^Rheumatology Department, Università Politecnica delle Marche, Jesi, Ancona, Italy; ^2^Medical Department, Pfizer, Rome, Italy

## Abstract

Chronic pain is nowadays considered not only the mainstay symptom of rheumatic diseases but also “a disease itself.” Pain is a multidimensional phenomenon, and in inflammatory arthritis, it derives from multiple mechanisms, involving both synovitis (release of a great number of cytokines) and peripheral and central pain-processing mechanisms (sensitization). In the last years, the JAK-STAT pathway has been recognized as a pivotal component both in the inflammatory process and in pain amplification in the central nervous system. This paper provides a summary on pain in inflammatory arthritis, from pathogenesis to clinimetric instruments and treatment, with a focus on the JAK-STAT pathway.

## 1. Introduction

Chronic pain is not only a critical symptom of rheumatic diseases but also has rather been defined as “a disease itself” and has wide biopsychosocial implications [1]. Pain in arthritis recognizes different mechanisms, including inflammation of the articular and periarticular structures, and both peripheral and central mechanisms are involved. With the disease progression, pain can derive from structural changes within the joint. These different aspects make difficult to correctly diagnose the type of pain and to treat it appropriately. Beyond the well-established analgesic therapies, new targets emerged over the last decade in the therapeutic approach to the multifactorial pain associated with inflammatory arthritis, such as the Janus kinase/signal transducer and activator of the transcription (JAK-STAT) pathway. This paper is an overview of the pathogenic mechanisms of pain in inflammatory arthritis, with a focus on the JAK-STAT signalling pathway. It attempts also to provide guidance on how to measure the impact of pain in rheumatic diseases and to treat it with old and new treatment approaches.

## 2. Neuroinflammatory Mechanisms of Pain

Pain is a multidimensional phenomenon, defined by the International Association for the Study of Pain (IASP) as “an unpleasant sensory and emotional experience associated with actual or potential tissue damage, or described in terms of such damage” [2]. Nociception is composed physiologically of four processes: transduction, transmission, modulation, and perception, in which transmission goes through sensory neurons, called nociceptors, which have high activation thresholds. Four main groups of pain are recognized, based on etiology: inflammatory, cancer, neuropathic, and central pain [3].

The nervous system communicates with the immune system [4], and inflammation at the site of the affected nerve is the common underlying mechanism between neuropathic and inflammatory pain. The central nervous system (CNS) elements involved in both development and maintenance of neuropathic pain are the microglia and the astrocytes. Mediators of neuropathic pain are cytokines and neurotrophic factors that are capable of activating neurons directly or via glial cells. To date, many substances have been identified as mediators of the neuropathic pain pathways. Moreover, both astrocytes and microglia can release proinflammatory cytokines able to activate glia and neurons expressing receptors for these molecules.

As recently reviewed by Busch-Dienstfertig and Gonzàlez-Rodrìguez [3], during the inflammatory process, an innate immune cascade occurs, yielding release of active factors from the blood and local and migrating inflammatory cells. Both pro- and anti-inflammatory cytokines are released. Among proinflammatory ones, tumor necrosis factor- (TNF-) *α* and interleukin- (IL-) 1 can directly sensitize nociceptive fibers and can activate different pathways, which in turn leads to the accumulation of more proinflammatory cytokines, that activate prostaglandin synthesis. TNF-*α* is synthesized by microglia, astrocytes, and some populations of neurons. TNF-*α* has several important functions in the CNS, including injury-mediated microglial and astrocyte activation, regulation of blood-brain barrier permeability, febrile responses, glutamatergic transmission, and synaptic plasticity. The mechanism in which IL-1*β* induces sensory neuronal sensitization to pain (through IL-1 receptor type-1 activation) is thought to involve tyrosine kinases as well as protein kinase C [5]. The great amount of prostaglandins accumulating in the injured tissue, as a consequence of this cascade, increases neuron sensitivity [6–9]. This “neuroinflammatory” environment activates in turn the glial cells in the brain and spinal cord, giving rise to the process of nociception [10].

Another element of communication between the immune and the nervous systems is the nerve growth factor (NGF), which mediates many of the activities exerted by cytokines as TNF-*α* and IL-1*β*. In fact, the NGF activates macrophages, inducing the release of multiple factors, including the NGF itself [11]. In summary, the process of pain involves several mechanisms mediated by both inflammatory and neural factors.

On the other hand, some cytokines contribute to anti-inflammatory and antinociceptive processes: IL-10 is a powerful anti-inflammatory cytokine, as shown in animal models of chronic pain [12], while IL-1*β* demonstrated to stimulate the synthesis of opioid receptors in dorsal root ganglia neurons [13]. Moreover, the immune cells present in inflamed tissues also contain opioids [14], whose release is dependent on proinflammatory cytokines, such as IL-1*β* and IL-6 [15, 16]. Glial cells can even release anti-inflammatory cytokines.

## 3. A Great Amplifier of Chronic Pain and Inflammation: Obesity

A large volume of evidence points to the concurrence of obesity and pain complaints [17]. Severe obesity in the elderly doubles the likelihood of having chronic pain [18]. Similarly, obesity appears to be a risk factor for developing neuropathic pain [19]. Research suggests that obesity may be characterized by a low-grade chronic inflammatory state as reflected by elevated levels in many inflammatory markers in the serum, such as IL-6 and C-reactive protein (CRP) [20–22]. Macrophage accumulation in adipose tissues has also been demonstrated in obese humans [23] and is known to play an important role in production and release of these inflammatory mediators [24]. Adipocytes, as well as providing an energy store for the body, are involved in the regulation of inflammatory processes and autoimmunity. It is known that adipocytes secrete approximately 50 different adipokines. Among these, the most well known are the following: leptin, visfatin, resistin, chemerin, and adiponectin [25, 26]. Leptin is considered a proinflammatory adipokine in immune cells and joint cells, although its role in animal models of arthritis remains unclear, with contrasting results [27]. Moreover, leptin exhibits similar structural and functional characteristics to the IL-6 cytokine family and increases inflammation by regulation of Th1-dependent response and production of proinflammatory cytokines by macrophages such as TNF-*α*, IL-6, and IL-12. Visfatin, also called nicotinamide phosphoribosyltransferase (Nampt), is a proinflammatory adipokine in immune and joint cells, and its blockade reduces arthritis severity similar to that of anti-TNF drugs. Resistin exists in two forms: as a hexamer or the more bioactive trimer. In rheumatoid arthritis (RA), it is associated with increased inflammation, particularly by the acute-phase reactant IL-1Ra antagonizing IL-1*β*, joint destruction, and glucocorticosteroid utilization [28]. Additionally, resistin may act as a marker of inflammation in other rheumatic disorders such as systemic lupus erythematosus (SLE) and systemic sclerosis (SSc) [29]. Chemerin is another active protein exhibiting two opposite actions. On one hand, it intensifies inflammation and stimulates chemotaxis of dendritic cells, macrophages, and NK cells to inflammation sites. Nevertheless, it inhibits production of inflammatory mediators and proinflammatory cytokines (TNF-*α* and IL-6) and stimulates adiponectin synthesis. Adiponectin has several isoforms and is involved in the pathogenesis of RA. There are studies indicating the activity of this adipokine in joints. It increases inflammation in joints, has a proinflammatory effect on chondrocytes, and contributes to destruction of the cartilage [30].

## 4. The JAK-STAT Pathway in Pain Modulation

Intracellular pathways are critical to immune cell activation, proinflammatory cytokine production, and cytokine signalling. JAKs are constitutively associated with many cytokine receptors. The binding of cytokines to a receptor associated with JAKs leads to the tyrosine phosphorylation of the receptor and generates a docking site for STATs. The STATs are thus phosphorylated and translocate to the nucleus where they may activate transcription of several genes [31].

In this complex milieu of products and cells, the JAK-STAT pathway is involved in the production of both pronociceptive and anti-inflammatory cytokines [3], playing an important role in the signalling pathway of neuropoietic cytokines and being also linked to the inflammatory response [32]. It has been reported to take on a role in nerve regeneration, in the inflammatory cascade activated by the microglia, and in the reaction of glial cells to CNS damage that leads to gliosis [33–35]. Reactive astrogliosis is a critical process for generating activated astrocytes. These cells may result in producing proinflammatory cytokines like TNF-*α*, IL-1*β*, and IL-6 and thereby in modulating dorsal horn pain processing.

In contrast to TNF-*α* and IL-1*β*, IL-6 through its specific receptor gp80 and the shared gp130 subunit, mainly activates the JAK-STAT transduction pathway. In addition, IL-6 may also signal via the mitogen-activated protein kinase cascade [36]. IL-6 induces JAK (JAK1 and JAK2) mediated phosphorylation of STAT1 and STAT3 proteins. IL-6 and STAT3 are key mediators of both chronic inflammation and joint destruction in RA [37]. In the CNS, STAT3 plays an essential role in IL-6 signalling. The blockade of the JAK-STAT3 activity prevented the strong expression of IL-6 and of other factors induced in the spinal cord after nerve lesion in rats and attenuated mechanical allodynia [38].

Another proinflammatory cytokine, IL-1*β*, is involved in the pathogenesis of the neuropathic pain component of RA regulated by the JAK-STAT pathway [39–41]. Inhibition of the JAK-STAT cascade blocks pro-IL-1*β* expression and IL-1*β* maturation, resulting in control of neuropathic pain. On the other hand, an anti-inflammatory and antinociceptive cytokine such as IL-4 carries out its effects via JAK-STAT [42–44].

Two other cytokines with predominantly proinflammatory action, namely, IL-12 and IL-18, also contribute to hyperalgesia by elevating the endothelin levels and by enhancing proinflammatory cytokines such as TNF-*α* [45]. Other molecules, as IL-13 and IL-10, share the JAK-STAT common pathway as a mechanism to exert their antinociceptive effect.

IL-10 has been shown to possess the most potent anti-inflammatory action, and its release downregulates the expression of IL-1*β*, IL-6, and TNF-*α*. It acts by downregulating proinflammatory genes, which leads to decreased expression of the abovementioned cytokines and their receptors and upregulation of their functional antagonists [46].

JAK-STATs showed to play a further role in maintaining neuropathic pain, by mediating astrocyte proliferation following nerve injury [47, 48]. To stop the astrocyte proliferative process may thus represent a therapeutic target for neuropathic pain after peripheral nerve injury. Another recent work showed that the microglial activity of the JAK-STAT3 proteins has effects on the functional properties of astrocytes and neurons [49], thus possibly participating in the remodelling of the spinal cord following peripheral nerve injury. Interestingly, the STAT3 signalling is implicated in astrogliogenesis from neural stem cells.

Conversely, the JAK-STAT pathway is of importance in the IL-4-induced upregulation of the opioid system [50–52]; in turn, endogenous and exogenous opioids stimulate IL-4 transcription in T cells [53, 54]. However, this strict connection between IL-4 and the opioid system is not the primary mechanism of IL-4 antinociceptive function, which is rather due to the inhibition of proinflammatory cytokines and factors [3, 55]. The relevance of IL-4 in pain was supported by an investigation of IL-4 deficiency in mice [56, 57].

In order to control the magnitude and duration of cytokine signalling, the JAK-STAT pathway is strongly regulated by different endogenous inhibitory mechanisms [36], including receptor internalization by vesicles followed by receptor degradation, posttranslational modifications of STAT proteins, and dephosphorylation by phosphatases (PTPs), as reviewed by Shuai [58].

In spite of this complex pathway and network of mediators, clinical trials with exogenous JAK inhibitors in the treatment of chronic inflammatory arthritis showed a significant positive impact on pain, as detailed below.

## 5. Pain in Inflammatory Arthritis

Patients with inflammatory arthritis commonly report pain as their most important problem, associated with psychological distress and impaired physical and social functioning. Moreover, pain is also a socioeconomic issue since it implies a significant decrease in work productivity and an increase in healthcare resource utilization, with a consequent significant impact on direct and indirect costs of rheumatic diseases [59, 60]. Pain plays an important role in health-related quality of life (HRQoL) in patients with RA. The Pain Management Task Force of the American College of Rheumatology (ACR) stated that “insufficient efforts were devoted to pain management” [61].

Pain is associated with disease activity [62–65], but it has been observed that although it is a marker of inflammation, its intensity poorly correlates with measures of inflammation [66, 67], and intense and disabling pain may persist even when the inflammatory disease is controlled [68]. Nonsteroidal anti-inflammatory drugs (NSAIDs) and biological and nonbiological disease-modifying antirheumatic drugs (DMARDs) are generally effective in relieving inflammatory pain symptoms. However, many patients continue to experience pain due to alterations in central pain regulation mechanisms, developing chronic widespread pain (CWP) [69, 70]. In this view, it is important to distinguish between inflammatory pain and sensitization in patients with rheumatic diseases, since therapies may be different. Mechanisms of peripheral and central RA pain in humans seem to be similar to those observed in animal models, where they have been widely investigated [71].

## 6. Peripheral Pain Mechanisms

Peripheral pain may be caused by articular alterations occurring during inflammatory joint diseases which can activate or sensitize primary afferent nociceptive neurons. In the RA synovium or synovial fluids, many algogens, cytokines, and chemokines are able to sensitize the peripheral nerves [72–76]. The peripheral sensitization is characterized by high spontaneous activity, a low threshold of activation of the nociceptive fibers, and increased responsiveness with local release of neuropeptides following stimulation [77]. The reiterative nociceptive stimulating action leads to sensitized spinal neurons. In humans with RA and in several animal models of inflammation, neutralization of TNF-*α* induces a rapid reduction of nociceptive neuronal activity in the afferent neurons. In the synovium and in the synovial fluids from patients with RA, opioids and anti-inflammatory cytokines have also been detected, but their ability to directly moderate arthritic joint pain has not been fully elucidated yet [78–82].

## 7. Central Pain Processing

Central pain processing is enhanced in RA [83]. Indeed, the degree of tissue inflammation or of articular damage is not the only predictor of the presence or the severity of pain. Under the category “central pain,” any dysfunction or pathologic condition of the CNS contributing to the development or maintenance of chronic pain is included. This concept also incorporates the psychosocial aspects of pain perception [83]. Central pain may also occur concomitantly with other symptoms that are centrally mediated, such as fatigue, insomnia, memory difficulties, and mood disturbances. Increased pain sensitivity regards not only inflamed joints but also remote, nonarticular sites, and reduced thresholds for pressure and thermal pain have also been reported in RA patients [84, 85]. Repeated painful stimuli seem also to have a role in the alterations of the diffuse noxious inhibitory control (DNIC), described as low functioning in patients with RA [82]. Functional magnetic resonance imaging (MRI) studies showed that patients with central pain states, when exposed to stimuli that are innocuous to healthy subjects, exhibit an increased neuronal activity in the brain regions devoted to pain processing, accounting for such a condition of diffuse hyperalgesia/pain augmentation. These pain-processing regions include the thalamus, the insular cortex (IC), the primary and secondary somatosensory cortex (SI and SII), the posterior cingulate cortex (PCC), and the anterior midcingulate cortex (ACC) [86]. Furthermore, it has been demonstrated that cerebral activity associated with evoked pain in RA is moderated by the psychological state [87]. Augmented pain processing has been associated with low mood, and such an association seems to be due to changes in cortical opioid receptor binding [88].

## 8. Psychological Aspects in Pain

Pain exacerbates psychological distress, and distress in turn can augment pain [71]. Worse mental health and higher levels of depression and anxiety have been reported in RA patients [86, 89, 90]. Depression is demonstrated to be associated with inflammatory disease activity, physical disability, poor treatment outcome, sensitivity to pain and reported pain severity, and even early mortality [91]. Recent findings also highlight that these variables contribute to the overuse of health services. Pain is predictive of depression in RA patients [92], and patients with depression report worse RA [93]. It has been shown that psychological characteristics are better predictors of pain compared to measures of joint inflammation or damage [94]. As in healthy people, in RA patients, factors of acute and chronic psychological distress can enhance central pain processing [91, 95]. Psychological interventions, particularly the cognitive behavioral therapy, or pharmacological treatments can reduce depression and alter central pain processing in people with RA [96, 97]. Furthermore, the prevalence of chronic, noninflammatory pain syndromes such as fibromyalgia (FM) is higher (15–30% of individuals) among patients suffering from RA than that in the general population (2%) [98, 99], suggesting that pain and/or stress accompanying chronic rheumatic diseases may trigger a condition such as FM. Patients with inflammatory arthritis and FM are more likely to have a poorer HRQoL than those without FM [61, 100], and the presence and severity of FM in RA also influence the response to biologic and traditional DMARDs and predict worse pain and functional prognosis following arthroplasty and back surgery [83].

**Figure 1:**
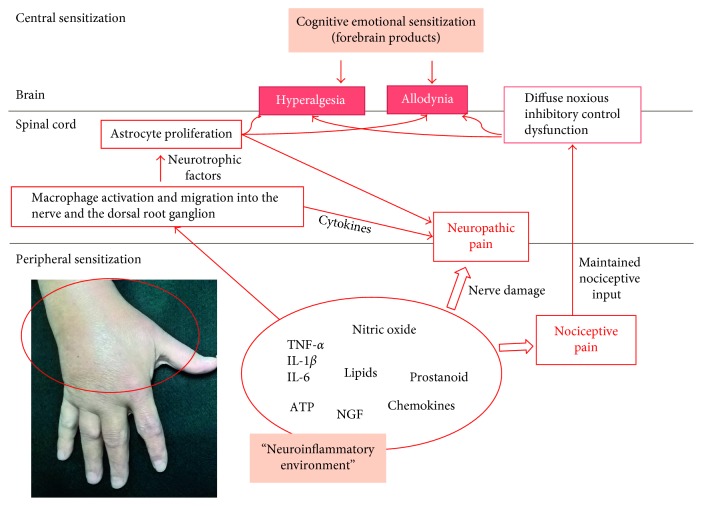
Peripheral and central mechanisms of pain sensitization in inflammatory arthritis. Synovitis can induce the production of molecules responsible for both nociception and neuropathic pain (through the damage of the nerves and the recruitment of macrophages in the nerves themselves and in the dorsal root ganglion). Repeated nociceptive stimuli can modify the function of the diffuse noxious inhibitory control (DNIC), with augmented pain perception as a consequence. Inflammatory cells stimulate, at the level of the central nervous system, the glial cell proliferation. Glial cells in turn provoke neural alterations responsible, at least in part, for hyperalgesia and allodynia. Pain sensitization is also strongly influenced by the psychological baggage.

Figure 1 depicts the actors involved in the supposed mechanisms of pain sensitization in inflammatory arthritis.

## 9. How to Measure Chronic Pain?

Individuals with chronic pain are frequently observed in rheumatological clinical practice, and the appropriate pain assessment may be crucial for successful pain management. As we described in a previous work [101], pain assessment is an interactive process that involves several actors: patients and their families, nurses, physicians, and other health professionals; pain assessment should include adequate diagnostic appraisal, physical evaluation, and a review of the patient's physical environment, of the psychosocial features, and of medical and surgical procedures. In any case, self-reporting scales remain the primary source, facilitating reassessments and follow-up. Different uni- and multidimensional pain measurement scales are available, none of which is suitable for all patients [102–104]. Moreover, novel instruments and healthcare monitoring systems based on information and communication technology have been developed to assess chronic musculoskeletal pain [101].

Some validated scales and questionnaires are commonly used in RA [70], including the Visual Analogue Scale (VAS), the Verbal Rating Scale (VRS), the Numerical Rating Scale (NRS), and the Faces Pain Rating Scale.

When a simple, one-item instrument is not enough to adequately measure pain [102], more comprehensive instruments combining measures of different dimensions of pain are needed [104]. However, these are often long tools with poor patient compliance. The most widely used multidimensional generic pain scales are the McGill Pain Questionnaire (MPQ) and its Short-Form (SF-MPQ), the Brief Pain Inventory (BPI), the Chronic Pain Grade Questionnaire (CPGQ), and the West Haven-Yale Multidimensional Pain Inventory (WHYMPI) [105–108]. A disease-specific scale to evaluate pain in patients with RA is the Rheumatoid Arthritis Pain Scale (RAPS) [109]. The painDETECT questionnaire, developed to measure noninflammatory and neuropathic pain, has been investigated in patients with different musculoskeletal pain conditions, including RA. The questionnaire is structured around nine items, two of which concern the temporal and spatial (radiating) characteristics of the individual pain pattern, and the other seven are weighted on sensory descriptors [110].

However, among unidimensional assessment tools, counting the number of swollen (SJC) and/or tender joints (TJC) remains the most specific quantitative clinical method to evaluate and monitor the status of patients with inflammatory arthritis [111]: namely, the SJC quantifies the inflammation, while the TJC better quantifies the amount of pain. Joint pain may be measured by the “rule of thumb” (i.e., pain at rest induced by a thumb and index pressure that whitens the examiner's nail bed) or by quantifying the pain on motion for the shoulder, tarsal, and hip joints. The number of joints considered can vary from 28 to 80, with different relative weights.

The domain “pain” is predominant also in assessing HRQoL in patients with RA. It strongly influences the scores of disease-specific scales, such as the Arthritis Impact Measurement Scales (AIMS) and AIMS2 [112, 113], and well-known generic scales, such as the European Quality of Life-5 Dimensions (EQ-5D) and Medical Outcomes Study (MOS) 36-Item Short-Form Health Survey (SF-36) [114, 115].  Table 1 summarizes the available scales to measure pain in patients with RA.

Disease-specific HRQoL tools are available for the majority of rheumatic diseases, and pain takes on a pivotal role in all of them [116–124]. Many of these instruments are patient-reported outcomes (PROs). PROs are gaining increasing importance in medicine [125]. Of particular interest is the easy applicability of PROs in the remote monitoring of patients through the web-based technologies [126, 127].

## 10. Effect of Pain on Disease Assessment

Studies showed that pain has a significant impact on the patient's assessment of RA disease activity. In a study analyzing 7,028 RA patients from the Quantitative Patient Questionnaires in Standard Monitoring of Patients with RA (QUEST-RA) database [128], pain emerged as the single most important determinant of the patient's global assessment of disease activity (PtGA). Another study, in 646 RA patients of an outpatient clinic starting methotrexate treatment, also concluded that pain was the major determinant of PtGA scores [63]. Pain contributes less to the physician's global assessment of disease activity (PhGA) than to PtGA: in the QUEST-RA study, pain was the fourth most important determinant of PhGA, after SJC, erythrocyte sedimentation rate, and TJC [66], while in the outpatient study, pain was second only to SJC among PhGA determinants [63]. In both studies, pain was one of the most significant predictors of discordance between PtGA and PhGA, being crucial in patients' “disease experience,” while often underestimated by clinicians. Other cross-sectional studies indicate that the presence of CWP or FM may significantly affect the assessment of disease activity in RA [84, 128–131], especially when subjective measures are used. Collectively, these results suggest that physicians should better assess the impact of pain on their patients but also has to consider the effect of coexisting central pain when using composite disease measures of disease activity in inflammatory arthritis.

## 11. Pharmacological Treatment of Pain in Inflammatory Arthritis

Recommendations for the pharmacologic management of pain in inflammatory arthritis have been recently published by the “3e (evidence, expertise, and exchange) initiative,” involving 17 nations [132]. The authors based their recommendations on Cochrane Database and other systematic reviews [133–138]. The main treatment options include (i) nonsteroidal anti-inflammatory drugs (NSAIDs) and acetaminophen as the first-line therapy, (ii) NSAIDs + acetaminophen or an alternative NSAID second-line therapy, and (iii) weak opioids when NSAIDs and acetaminophen have failed or are contraindicated. Antidepressants may be used as adjuvant therapy, but their analgesic role in inflammatory arthritis is still controversial [139]. The 3e initiative recommends gabapentin and pregabalin as potential adjuvant treatments that were shown to reduce the release of neurotransmitters in peripheral pain syndromes. The 3e initiative authors strongly recommend to take into consideration the type of pain and of arthritis, the presence of comorbidities, the addictive potential of the medication, and the patient's preference when choosing the most appropriate pain treatment [132].

A Cochrane systematic review of the existing literature included a majority of studies dated before 1990 [140], and efficacy data beyond six weeks of treatment duration as well as in comorbid patients are scarce [141–145]. There is a need for new large and long-term trials, including also patients with comorbidities to optimize the use of analgesics and adjuvant therapy in RA patients. In Table 2 are listed the main Cochrane systematic reviews about pain treatment in patients with inflammatory arthritis.

Traditional DMARDs, such as methotrexate, sulphasalazine, and leflunomide, reduce joint pain while suppressing inflammation over several weeks, maintaining these effects over months, and their rapid introduction is recommended by current guidelines, preferably in combination. Many common DMARDs may have some of their adverse effects potentiated by specific analgesic medications; however, a Cochrane systematic review concluded that NSAIDs in combination with methotrexate are generally safe, though recommending appropriate monitoring and avoidance of ASA [146]. In the presence of active disease and pain that is inadequately controlled by methotrexate, addition of a biologic agent may be useful [147]. Biologic DMARDs include TNF-blocking agents (infliximab, adalimumab, golimumab, certolizumab pegol, and etanercept); rituximab, an anti-CD20 B-cell-depleting monoclonal antibody (MoAb); tocilizumab which is a IL-6 receptor-blocking MoAb; the IL-1 receptor antagonist anakinra; and the T-cell activation inhibitor abatacept. Second-generation drugs include the anti-IL-17 MoAbs secukinumab and ixekizumab; brodalumab, a MoAb blocking the IL-17 receptor; selective small-molecule inhibitors of intracellular signal transduction pathways, such as the JAK pathway (tofacitinib and baricitinib); and the spleen tyrosine kinase (fostamatinib). Biologic agents reduce joint pain in RA by reducing inflammation, decreasing peripheral and central sensitization, and preventing long-term joint damage [70, 71].

A growing interest for the treatment of patients suffering from chronic diseases is directed towards pleiotropic natural products. Many substances, such as omega-3 polyunsaturated fatty acids (n-3 PUFA), curcumin, resveratrol, theanine, theaflavin derivatives, and *α*-lipoic acid, can be incorporated into pharmacotherapies to improve therapy outcomes. These compounds, when combined with pharmaceutical drugs, showed improved efficacy and safety in preclinical and clinical studies of neuropathic pain.

Curcumin, in particular, the primary active ingredient of turmeric (*Curcuma longa*), has been demonstrated to possess anti-inflammatory, antiosteoclastogenic potential, and antiarthritic properties [148–150]. Treatment with curcumin seems to lessen mechanical allodynia and thermal hyperalgesia through downregulation of TNF-*α* and TNF-*α* receptor 1 expression [151]. On the rat model of neuropathic pain, curcumin can markedly alleviate nerve injury-induced neuropathic pain. This analgesic effect may be attributed to the inhibition of astrocyte hypertrophy in the spinal dorsal horn and phosphorylation of the ERK signalling pathway [152]. Moreover, curcumin can inhibit the secretion of TNF-*α*, IL-1*α*, IL-6, and nitric oxide, which can further promote the activation of astrocytes.

## 12. Effect of JAK Inhibitors on RA Pain

Many of the key cytokines use the JAK-STAT pathway to exert their effects rendering them amenable to therapeutic blockade with JAK inhibitors. Given the apparent pathogenic role of a variety of cytokines like IL-6, IL-12, IL-23, interferons, and GM-CSF in RA and other autoimmune diseases, the ability of JAK inhibitors to block such cytokines is likely a major aspect of their mechanism of action. Next to the already existing tofacitinib and baricitinib, a number of other JAK inhibitors are currently in development for the management of RA, with differing in vitro specificities towards the various members of the JAK family. Mechanistically, tofacitinib blocks common cytokines including IL-2, IL-4, IL-7, IL-9, IL-15, and IL-21, all of which are signals through JAK3. In addition, it blocks JAK1, which would result in inhibition of the gp130 family including IL-6 and IL-11 as well as type II cytokine receptor family such as IFN-*α*/*β*, IFN-*γ*, and IL-10 [153]. Tofacitinib is so far the most extensively studied JAK inhibitor, and its effect on the clinical and laboratory measures of RA (ACR20, ACR50, ACR70, DAS28, etc.) is well documented in reviews and meta-analyses [154–161]. Its impact on PROs is also provided in almost all published efficacy studies or reported in specific publications [162–164] and has been recently reviewed by Boyce and colleagues [165]. The results show a significant reduction in RA patients' assessment of pain with tofacitinib compared to placebo. Some patients report pain relief within the first 24 hours of JAK inhibitor administration, well before a demonstrable effect on inflammation [164]. Data on the patients' assessment of pain and/or PtGA of the disease are available from further seven clinical studies [166–172]. Tofacitinib, administered 5 mg bid, was associated with a 45%–54% improvement in the patients' assessment of pain and a 44%–60% improvement in PtGA, while placebo resulted in less improvement (29% for pain and 39% for PtGA) [165]. Overall, it can be concluded that patients' assessment of pain and disease demonstrate that tofacitinib is more effective than placebo.

Baricitinib blocks JAK1 and JAK2 in a highly selective way, interrupting the pathways of several cytokines considered important in RA pathogenesis [173]. In the phase III RA-BUILD clinical trial, baricitinib was associated with clinical improvement and inhibition of progression of radiographic joint damage in patients with RA and an inadequate response or intolerance to conventional synthetic DMARDs [174]. In the phase III RA-BEACON study in RA patients, baricitinib 4 mg significantly improved pain and other PROs over 24 weeks [172].

## 13. Conclusions

We are acutely aware that pain is a major component of many rheumatic diseases, arising from physiological interactions involving the peripheral and central nervous systems. Peripheral signalling, in the form of nociceptive inputs, such as peripheral structural damage and/or inflammation, is mainly implicated in acute pain, whereas inputs from the CNS are responsible for chronic pain. The origin of CWP in patients with arthritis is complex, involving genetic and/or familial predisposition and psychological factors. It is worth underlining that, from the patient's perspective, pain in inflammatory arthritis is reported as the most important problem, significantly affecting HRQoL and working ability. Furthermore, from the clinicians' perspective, pain impacts on disease assessment and treatment choices. Pain has been demonstrated to be the major determinant in PtGA but not in PhGA [63, 66]. Therefore, we strongly recommend, for effective care of RA, to pay greater attention to the “disease experience” of patients, particularly pain, and to perform accurate pain measurement.

A range of pharmacological analgesics, immunomodulatory agents, serotonin-norepinephrine reuptake inhibitors, and physical and psychological interventions are available to help relieve pain in rheumatic patients. The role of the JAK-STATs in the modulation of both inflammatory and neuropathic pain drew attention to the possible effect of JAK inhibitors, specifically on RA-associated pain. The results are promising, but more experimental and clinical studies and trials of longer duration are required to elucidate the possible role of these molecules in the modulation of pain, as well as in the overall relief of RA burden. Pain-relieving drugs have different mechanisms of action, and we hope that it will be possible, in the next future, to target specific drugs for individual patients in order to improve the outcome for patients with chronic pain.

## Figures and Tables

**Table 1:** Scales to measure all the aspects of pain in patients with rheumatoid arthritis.

Pain scales		Health-related quality of life scales		Pain location scales	Site-specific scales
Unidimensional	Multidimensional	Generic	Disease specific		
Verbal Rating Scale (VRS)	McGill Pain Questionnaire (MPQ)	36-Item Short-Form Health Survey (SF-36)	Arthritis Impact Measurement Scales (AIMS)	Formal Joint Count	Western Ontario and McMaster Universities Osteoarthritis Index (WOMAC)
Visual Analogue Scale (VAS)	Short-Form MPQ (SF-MPQ)	European Quality of Life-5 Dimensions (EQ-5D)	Arthritis Impact Measurement Scales 2 (AIMS2)	Regional Pain Scale (RPS)	Hip Disability and Osteoarthritis Outcome Score (HOOS)
Numerical Rating Scale (NRS)	Brief Pain Inventory (BPI)	Sickness Impact Profile (SIP)			Disabilities of the Arm, Shoulder, and Hand (DASH) Questionnaire
Faces Pain Rating Scale	Chronic Pain Grade Questionnaire (CPGQ)	Nottingham Health Profile (NHP)			
Thermometer Pain Scale (TPS)	West Haven-Yale Multidimensional Pain Inventory (WHYMPI); Rheumatoid Arthritis Pain Scale (RAPS)				

**Table 2:** Principal Cochrane systematic reviews on pain management in inflammatory arthritis.

Topics	Main conclusions
Opioids (Whittle et al. [133])	(i) Limited evidence that weak oral opioids may be effective analgesics for some patients with rheumatoid arthritis, but adverse effects are common and may offset the benefits of this class of medications.
	(ii) Insufficient evidence to conclude regarding the use of weak opioids for longer than six weeks or the role of strong opioids.
Neuromodulators (Richards et al. [135])	(i) Weak evidence that oral nefopam, topical capsaicin, and oromucosal cannabis are all superior to placebo in reducing pain in rheumatoid arthritis patients.
	(ii) Capsaicin could be considered as an add-on therapy for patients with persistent local pain and inadequate response or intolerance to other treatments.
Antidepressants (Richards et al. [139])	(i) Insufficient evidence to support the routine prescription of antidepressants as pain modulators in rheumatoid arthritis patients since no reliable conclusions about their efficacy can be gathered from eight placebo randomized controlled trials.
Pain management in rheumatoid arthritis and cardiovascular or renal comorbidity (Marks et al. [141])	(i) Absence of specific evidence in rheumatoid arthritis.
	(ii) Guidelines recommend that nonsteroidal anti-inflammatory drugs should be used with caution in the general rheumatoid arthritis population, with the need of extra vigilance in patients with established cardiovascular disease or risk factors.
	(iii) Guidelines regarding the use of nonsteroidal anti-inflammatory drugs and opioids in moderate-to-severe renal impairment should also be applied to the rheumatoid arthritis population.
Pain management in inflammatory arthritis and gastrointestinal or liver comorbidity (Radner et al. [142])	(i) Scarce evidence to guide clinicians about how gastrointestinal or liver comorbidities should influence the choice of pain therapy.
	(ii) Nonsteroidal anti-inflammatory drugs should be used cautiously in patients with inflammatory arthritis and a history of gastrointestinal comorbidity since the evidence that they may be at increased risk is consistent.
Combination therapy for pain management in inflammatory arthritis (Ramiro et al. [140])	(i) Insufficient evidence to agree upon the value of combination therapy over monotherapy.
	(ii) No studies have addressed the value of combination therapy for patients with inflammatory arthritis having persistent pain despite optimal inflammation control.
